# Molecular Characterization of Esophageal Squamous Cell Carcinoma Using Quantitative Proteomics

**DOI:** 10.3390/cancers15133302

**Published:** 2023-06-23

**Authors:** Kiran K. Mangalaparthi, Krishna Patel, Aafaque Ahmad Khan, Bipin Nair, Rekha V. Kumar, Thottethodi Subrahmanya Keshav Prasad, David Sidransky, Aditi Chatterjee, Akhilesh Pandey, Harsha Gowda

**Affiliations:** 1 Institute of Bioinformatics, International Technology Park, Bangalore 560066, India; 2 Amrita School of Biotechnology, Amrita Vishwa Vidyapeetham, Kollam 691001, India; 3 Department of Laboratory Medicine and Pathology, Mayo Clinic, Rochester, MN 55905, USA; 4 Department of Pathology, Kidwai Memorial Institute of Oncology, Bangalore 560066, India; 5 Center for Systems Biology and Molecular Medicine, Yenepoya Research Centre, Yenepoya (Deemed to be University), Mangalore 575018, India; 6 Department of Oncology, The Sidney Kimmel Comprehensive Cancer Center, Johns Hopkins University School of Medicine, Baltimore, MD 21287, USA; 7 Department of Otolaryngology and Head & Neck Surgery, Johns Hopkins University School of Medicine, Baltimore, MD 21205, USA; 8 Manipal Academy of Higher Education, Manipal 576104, India; 9 Center for Individualized Medicine, Mayo Clinic, Rochester, MN 55905, USA; 10 Center for Molecular Medicine, National Institute of Mental Health and Neurosciences, Hosur Road, Bangalore 560029, India

**Keywords:** esophageal squamous cell carcinoma, quantitative proteomics, cell cycle regulation, ER stress, endoplasmic reticulum-associated degradation (ERAD), SYVN1

## Abstract

**Simple Summary:**

In this study, we carried out global proteomic profiling of esophageal squamous cell carcinoma and adjacent non-neoplastic esophageal tissue to identify putative biomarkers and targets. We identified several differentially expressed proteins including PDPN, TOP2A, POSTN and MMP2 that were overexpressed in ESCC. We also identified overexpression of SOX2, TP63, IGF2BP2 and RNF13 that are encoded by 3q26 region, a known hotspot region in ESCC. Synoviolin 1 (SYVN1), a protein involved in endoplasmic reticulum (ER)-associated degradation and other ER stress response proteins were overexpressed in ESCC. SYVN1 could be a potential therapeutic target in ESCC.

**Abstract:**

Esophageal squamous cell carcinoma (ESCC) is a heterogeneous cancer associated with a poor prognosis in advanced stages. In India, it is the sixth most common cause of cancer-related mortality. In this study, we employed high-resolution mass spectrometry-based quantitative proteomics to characterize the differential protein expression pattern associated with ESCC. We identified several differentially expressed proteins including PDPN, TOP2A, POSTN and MMP2 that were overexpressed in ESCC. In addition, we identified downregulation of esophagus tissue-enriched proteins such as SLURP1, PADI1, CSTA, small proline-rich proteins such as SPRR3, SPRR2A, SPRR1A, KRT4, and KRT13, involved in squamous cell differentiation. We identified several overexpressed proteins mapped to the 3q24-29 chromosomal region, aligning with CNV alterations in this region reported in several published studies. Among these, we identified overexpression of SOX2, TP63, IGF2BP2 and RNF13 that are encoded by genes in the 3q26 region. Functional enrichment analysis revealed proteins involved in cell cycle pathways, DNA replication, spliceosome, and DNA repair pathways. We identified the overexpression of multiple proteins that play a major role in alleviating ER stress, including SYVN1 and SEL1L. The SYVN1/SEL1L complex is an essential part of the ER quality control machinery clearing misfolded proteins from the ER. SYVN1 is an E3 ubiquitin ligase that ubiquitinates ER-resident proteins. Interestingly, there are also other non-canonical substrates of SYVN1 which are known to play a crucial role in tumor progression. Thus, SYVN1 could be a potential therapeutic target in ESCC.

## 1. Introduction

Esophageal squamous cell carcinoma (ESCC) accounts for 90% of esophageal cancer cases, with a high incidence in Asian countries, including India [1]. It arises from the upper two-thirds of the esophagus, whereas the other histological subtype, esophageal adenocarcinoma, arises from the lower portion of the esophagus. The incidence of ESCC is influenced by many factors such as lifestyle, race and sex, environmental factors such as tobacco usage and alcohol consumption. The development of ESCC is multifactorial that involves transforming normal squamous epithelium to low-grade intraepithelial neoplasia, high-grade intraepithelial neoplasia, and then to invasive carcinoma [2]. Chemotherapeutic regimen involving cisplatin and 5-fluorouracil remains a mainstay for treating inoperable cases of ESCC. Hence, the identification of novel biomarkers is essential for early diagnosis and clinical management of ESCC.

Several studies have been carried out in recent years to investigate global genomic alterations associated with ESCC. These studies have revealed *TP53*, *NOTCH1*, *PIK3CA*, *FAT1*, *NFE2L2* as some of the significantly mutated genes in ESCC tumors across different ethnic cohorts [3,4,5,6]. Recurrent copy number variations in ESCC include amplification in 3q24, 11q13, 8p23.3 regions, and deletion of 9q21.3, which harbors several key genes that have a role in tumorigenic mechanisms [7]. Multiple studies investigated the global transcriptome profiles of ESCC [8,9]. However, mRNA expression often does not accurately reflect protein expression changes. Therefore, gene expression alone fails to reflect the functional state of tumors [10,11,12]. Investigating molecular alterations at the proteomic level will enable a better understanding of the molecular mechanisms that drive ESCC and result in the identification of novel protein biomarkers. Mass spectrometry-based proteomics has taken center stage in quantitative proteomic analysis due to improved sample preparation strategies and instrumentation enabling high throughput analysis of protein expression patterns [13,14,15]. Our group has previously employed iTRAQ-based quantitative proteomics strategy on pooled tumor and normal tissue samples and identified biomarkers of ESCC, including PLEC1, PSAP, and PDIA4 [16]. Another study on quantitative tissue proteomic analysis of high-grade esophageal squamous intraepithelial neoplasia (HGIN), a precancerous lesion of ESCC, identified proteins IGF2BP3, NUP188, and MOCS3 overexpressed in HGIN, suggesting their potential involvement in the development of ESCC [17]. Several other studies have employed quantitative proteomics strategies to characterize differential protein expressions in ESCC [16,18,19]. Recently, Zhu et al. applied label-free quantitative proteomic analysis in ESCC to identify differentially expressed proteins. They further validated overexpression of prothymosin alpha (PTMA) by quantitative dot blot and immunohistochemistry [20]. In this study, we employed high-resolution mass spectrometry-based quantitative proteomic analysis on 12 pairs of ESCC tumors and paired normal tissues. Our study identified several proteins that can serve as potential markers and are potential candidates for further validation in ESCC.

## 2. Materials and Methods

### 2.1. Sample Collection

Twelve pairs of tumor and adjacent normal tissue samples were collected from Kidwai Memorial Institute of Oncology, Bangalore, India, with the institutional review board’s prior approval. Informed consent forms were obtained from all the patients before the sample collection. All the samples were stored at −80 °C until further processing. Sample details are provided in Supplementary Table S1.

### 2.2. Protein Extraction, Digestion, and TMT Labelling

Tissue samples were homogenized into a fine powder using liquid nitrogen grinding. Samples were lysed in 4% SDS lysis buffer in 50 mM TEABC and subjected to heating at 90 °C for 5 min. They were sonicated for 3 cycles at 40% amplitude on ice and heated again at 90 °C for 5 min. Protein estimation was performed using BCA protein assay kit. Equal amounts of protein from each sample were reduced using 5 mM dithiothreitol and incubated at 60 °C for 20 min. Samples were cooled and alkylated by incubation in 10 mM iodoacetamide for 15 min in the dark. Five volumes of chilled acetone were added to precipitate proteins and kept at −20 °C overnight. Pellets were washed with 80% acetone once and centrifuged at 14,000 RPM for 15 min. Pellets were suspended in 6 M Urea in 50 mM TEABC buffer and subjected to LysC digestion (1:75 enzyme to protein ratio) for 4 h at 37 °C. Further digestion was carried out using Trypsin at 1:75 (enzyme to protein ratio) and incubated overnight at 37 °C. Samples were acidified with 1% formic acid and cleaned using Sep-Pak C_18_ columns. Next, 50 µg peptides from all samples were pooled and used as bridging channels between the experiments. A total of 12 tumor and adjacent normal tissue samples were distributed between 3 tandem mass tag (TMT) experiments (4 tumors and 4 adjacent normal tissues per experiment) with the pooled sample using the other 2 channels for each TMT 10plex reaction. From each sample, 200 µg of peptide digest was labeled with TMT 10plex reagent as per manufacturer’s instructions. After labeling, all the samples in each TMT set were pooled and cleaned using Sep-Pak C_18_ columns. The samples were fractionated using basic pH RPLC fractionation method on Agilent 1260 HPLC system. Briefly, the sample was suspended in solvent A (5 mM ammonium formate, pH 10) and separated on Zorbax C_18_ column using a linear gradient of 5% solvent B (90% acetonitrile, 5 mM ammonium formate, pH 10) to 60% over 90 min. Finally, the fractions were pooled and reduced to 12 fractions, speed vac concentrated and cleaned using C_18_ stage tips.

### 2.3. LC-MS/MS Analysis and Database Searching

All the fractions were analyzed on Q-Exactive HF-X mass spectrometer (Thermo Scientific, Bremen, Germany) using a front-end Dionex Ultimate RSLC 3000 system (Thermo Scientific, Bremen, Germany). The solvent system used in LC includes solvent A (0.1% formic acid in MS grade water) and solvent B (80% acetonitrile, 0.1% formic acid). Peptide mixture was loaded on a trap column (Thermo Scientific, Bremen, Germany, Acclaim PepMap 100, 75 µm × 2 cm, 3 µm C_18_ 100 A°) and separated with a linear gradient of 8% to 35% solvent B on an analytical column (Thermo Scientific, Bremen, Germany, Acclaim PepMap RSLC, 75 µm × 50 cm, 2 µm C_18_). The total run time was 130 min with 15 min initial equilibration step in each run. The precursor ions were surveyed in Orbitrap mass analyzer with 350–1600 *m/z* mass range. The MS scans were recorded using 120,000 resolution at 200 *m*/*z* with 30 ms maximum injection time. MS/MS scans were triggered using data-dependent topN mode in which top 15 precursor ions from the MS scans were isolated using quadrupole mass filter and fragmented using high energy collision-induced dissociation (NCE 32%). An isolation width of 1.2 *m/z* was used to minimize the co-isolation of precursor ions. Fragmented product ions were analyzed again in Orbitrap mass analyzer at 50,000 resolution and 100 ms maximum injection time. Dynamic exclusion of 30 s, charge state filter and monoisotopic precursor selection were enabled. Polysiloxane ion 445.12002 *m/z* was used as an internal calibrant during the run. All the fractions were analyzed in triplicate.

All the raw files were analyzed using Proteome Discoverer software (Version 2.2) for protein identification and quantification. Raw data were searched against the human UniProt protein database using Sequest and Mascot search algorithms with the following parameters: trypsin as enzyme with two missed cleavages, oxidation at methionine and acetyl modification at protein N-terminus as variable modification, carbamidomethylation at cysteine and TMT modification at N-terminus and lysine as static modifications. Precursor mass tolerance of 10 ppm and fragment mass tolerance of 0.02 Da were allowed during the search. The reporter ions’ intensity was obtained using an integration tolerance of 30 ppm around the reporter masses. For reliable quantitation, only PSMs filtered for a co-isolation threshold of 70%, and an average S/N value of 10 were considered for protein quantitation. Protein identifications were filtered at 1% protein-level FDR using the software’s protein level FDR validator node.

### 2.4. Inter-Experiment Normalization of Quantitative Proteomics Data

Inter-experiment normalization of protein abundances was performed using internal reference scaling (IRS) method described by Plubell et al. [21]. An intra-experiment normalization was performed within the Proteome Discoverer software using total intensity-based normalization. The bridging channels for each protein were averaged and normalized across the three experiments to calculate each protein’s scaling factors. These scaling factors were multiplied with the abundances across all the samples in 3 experiments, and these IRS-normalized abundances were used in differential expression analysis.

### 2.5. Bioinformatics Data Analysis of Quantitative Proteomics Data

Bioinformatics data analysis was carried out using Perseus computational platform [22]. Differential expression analysis was carried out on logarithmized abundances using a 2-sample test with multiple hypothesis correction. Principle component analysis and heat map visualization was performed on log base 10 transformed data, without any scaling other than as described in Section 2.4, using MetaboAnalyst 5.0 (https://www.metaboanalyst.ca/ (accessed on 31 August 2022)) [23]. Pathway enrichment analysis of the differentially expressed proteins was performed by KEGG pathway analysis using the DAVID web server [24] separately for overexpressed and under expressed proteins, with proteins commonly identified in all three experiments used as background database. Protein–protein interaction networks were generated using the String database [25] and the networks were further visualized using Cytoscape application V3.8 [26].

## 3. Results

### 3.1. Global Proteomic Analysis of ESCC Tumors and Adjacent Normal Tissues

**Figure 1:**
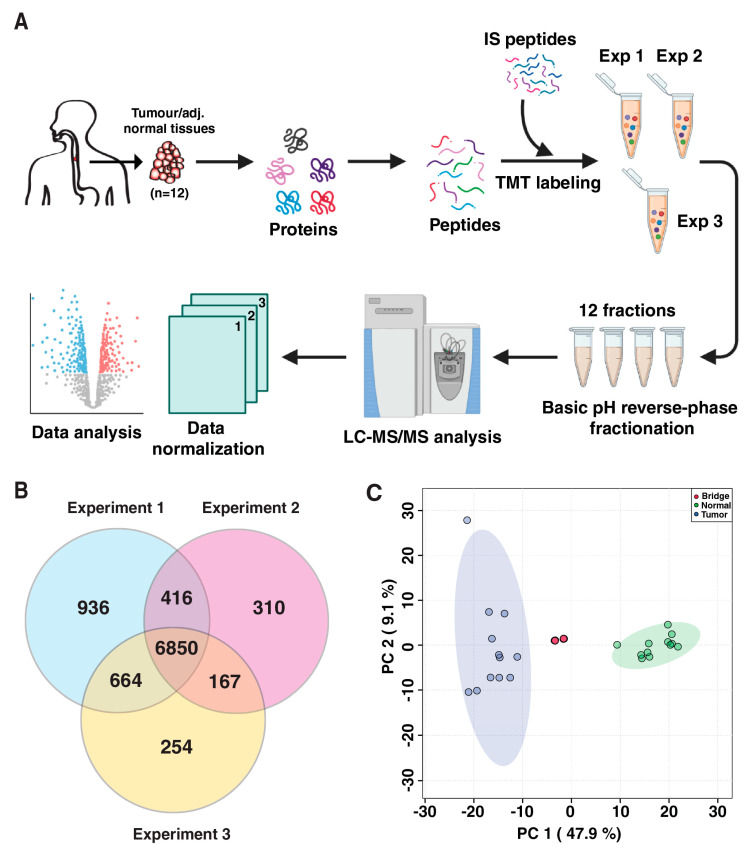
Overview of the quantitative proteomic analysis of ESCC tumor and adjacent normal tissues. (**A**) TMT-based workflow employed for the quantitative proteomics analysis. Twelve pairs of tumor and normal tissues were distributed across three TMT 10plex experiments with four pairs of samples in each experiment and two pooled internal standard peptides used for inter-experimental normalization. Raw data files were analyzed using Proteome Discoverer 2.2, and proteins were filtered at 1% protein-level FDR. (**B**) Overlap of all the proteins identified across three experiments shows 6850 proteins commonly identified in all three experiments. (**C**) Distinct segregation of ESCC tumor and normal tissues in 2-dimensional space using principal component analysis. TMT channels used for internal standard peptides across the three experiments were clustered together.

The experimental workflow followed in this study is depicted in Figure 1A. In total, 12 pairs of ESCC tumor and matched normal tissue samples were used for quantitative proteomic analysis using three multiplexed TMT 10plex labeling experiments. TMT-based quantitative proteomic analysis enables global proteome profiling for relative quantitation of proteins across multiple samples. This approach has been widely used in proteomics [27,28,29]. We identified 9597 proteins across all three experiments, including 6850 proteins that were commonly identified across all 12 samples (Figure 1B, Supplementary Table S2). In each experiment, fractions analyzed in technical triplicates showed a high Pearson correlation (r = ~0.95) between the replicates, indicating high technical reproducibility of the experiments. Further, a common bridging channel was used in duplicate in all three experiments to normalize reporter ion abundances across the experiments. The validity of normalization was evident based on correlation matrix (Supplementary Figure S1A). The principal component analysis (PCA) showed clear segregation of tumor samples from normal tissue samples, suggesting distinct protein expression changes between tumors and normal tissue samples (Figure 1C). Protein loadings from the PCA analysis are given in Supplementary Table S3. Tobacco use is the major risk factor associated with ESCC. However, we did not observe a significant difference in protein expression pattern between tobacco users and non-users using PCA. Furthermore, differential expression analysis using ANOVA showed no significant difference between tobacco users and non-users in our cohort (Supplementary Figure S1B). This is likely due to the small sample size used in our study.

### 3.2. Differential Expression Analysis between Tumor and Matched Normal Tissue Samples

**Figure 2:**
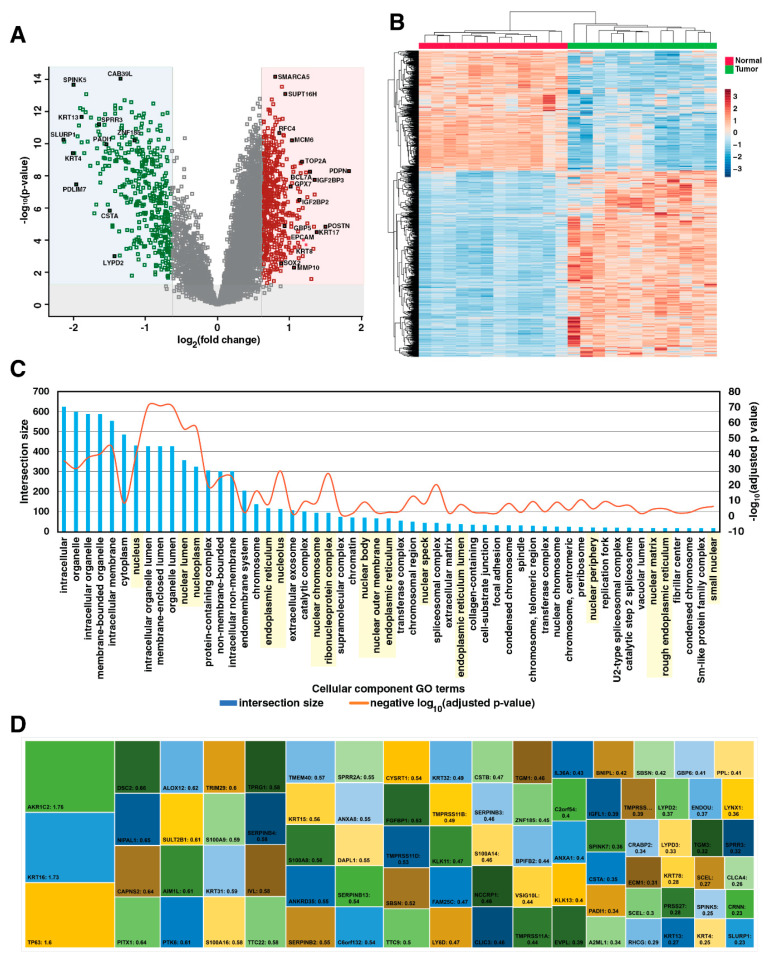
Differential expression analysis of 6850 proteins commonly identified in all three experiments. (**A**) Volcano plot depicting statistically significant differentially expressed proteins using 2- sample *t*-test with multiple hypothesis testing (fold change 1.5-fold, FDR q-value < 0.05). Overexpressed proteins are highlighted in red, and downregulated proteins are highlighted in green. (**B**) Unsupervised clustering of the differentially expressed proteins indicated distinct clusters of protein expression in tumors compared to adjacent normal tissues. (**C**) Gene ontology cellular component analysis of the differentially expressed proteins suggested enrichment of nuclear and endoplasmic reticulum-specific activities among the dysregulated proteins. These cellular components are highlighted in yellow color. (**D**) Tree map of the esophageal tissue-enriched proteins differentially expressed in ESCC. The size of the box indicates the fold change value of the differentially expressed protein.

Normalized reporter ion abundances of 6850 proteins common to all three experiments were considered for differential expression analysis. After a two-sample *t*-test with multiple hypothesis correction, 693 proteins were found to be significantly overexpressed, and 446 proteins were found to be significantly downregulated by ≥1.5-fold (Figure 2A, Supplementary Table S4). Hierarchical clustering of proteins revealed distinct proteomic alterations in ESCC tumor samples compared to normal tissues (Figure 2B). Proteins localized to endoplasmic reticulum and nucleus were significantly enriched among differentially expressed proteins (Figure 2C). We identified several proteins previously reported in ESCC including PDPN, POSTN, TOP2A, MMP12, UBE2C and PLAU, which were overexpressed and CRNN, SPRR3, SORBS2, RHCG, ECM1, and TGM3, which were downregulated. A comparison of different publicly available RNAseq datasets on ESCC revealed high concordance with differentially expressed proteins identified in our study (Supplementary Figure S2).

Downregulated proteins included several esophagus-specific proteins, including SLURP1, CRNN, KRT4, and KRT13 (Figure 2D). Terminal differentiation markers such as KRT4, KRT13, and IVL were downregulated, and KRT14, a marker of undifferentiated and proliferating keratinocytes, was overexpressed [30]. Among 251 proteins with high expression in the esophagus retrieved from the Human Protein Atlas [31], 79 proteins were downregulated and 3 proteins, TP63, KRT16, and AKR1C2, were overexpressed.

These proteins are involved in epithelial cell differentiation, tissue development, and peptidase activity regulation. Small proline-rich proteins (SPRR2A, SPRR3), keratins (KRT4, KRT13), TGM3, S100A8/S100A9 are downregulated proteins involved in the maintenance of squamous phenotypes [32]. This indicates the loss of the squamous phenotype and tissue uniqueness during esophageal squamous cell carcinoma progression, and these molecules could serve as good prognostic candidates for monitoring disease progression.

### 3.3. Genes Encoded by 3q24-29 Amplicon—A Hotspot Region in ESCC

**Figure 3:**
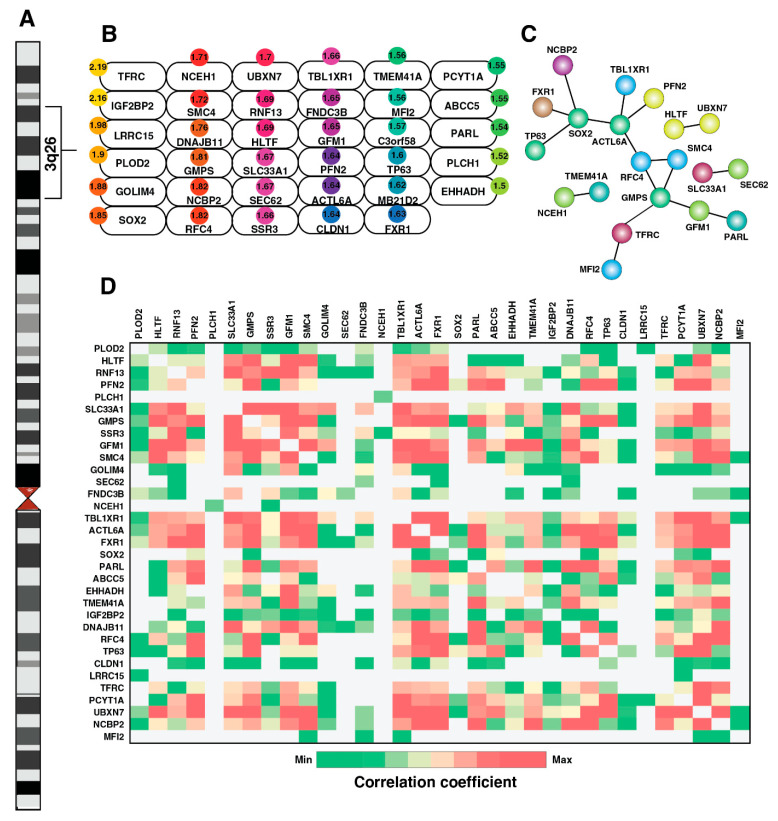
Mapping differentially expressed proteins in the 3q24-29 chromosomal hotspot in esophageal squamous cell carcinoma. (**A**,**B**) Schematic representation of Chr3 cytobands highlighting overexpressed proteins in ESCC tumor samples belonging to 3q24-29 chromosomal region. Circle on the top of each box indicates the fold change overexpression in tumor samples. (**C**) Protein–protein interaction network among the overexpressed proteins from 3q24-29 hotspot. (**D**) Gene expression correlation between pair of 3q24-29 genes identified using TCGA-ESCA dataset hosted on UALCAN platform.

Genomic copy number alterations were shown to be driver events in the tumorigenic process across several cancers. Our previous study on ESCC reported recurrent amplification of genes in the 3q region [6]. The 3q amplicon contains key oncogenic driver genes such as *TP63*, *SOX2*, *PIK3CA*, which are implicated in all squamous cell carcinomas, including ESCC [33]. Co-amplification of genes *SOX2* and *TP63* is uniquely seen in squamous cell carcinomas [34]. In this study, we observed statistically significant enrichment of differentially expressed proteins belonging to the 3q (*p*-Value < 2.97 × 10^6^), 3p and 1q (*p*-Value 0.003) regions. In concordance with copy number alterations reported by several studies, we identified the overexpression of SOX2, TP63, and ACTL6A belonging to the 3q26 amplicon (Figure 3A,B). Protein–protein interaction showed strong connectivity among proteins belonging to this region (Figure 3C). Using the publicly available TCGA-ESCA dataset, protein and transcript expression were found to be positively correlated among several genes encoded by the 3q24-29 region, indicating copy number amplifications result in protein-level overexpression (Figure 3D). RNF13 is a novel candidate not reported previously in ESCC. We also observed a positive correlation between copy number alterations of TP63, IGF2BP2, and RNF13 in the TCGA-ESCA dataset. IGF2BP2, along with IGF2BP1 and IGF2BP3, belong to the IMF family of proteins, which play an important role in post-transcriptional regulation [35]. In addition to IGF2BP2, we also observed the overexpression of IGF2BP1 and IGF2BP3 in our study. Though several substrates of IGF2BP1 and IGF2BP3 are known in cancers, including ESCC, the downstream mRNAs regulated by IGF2BP2 are largely unknown. RNF13 belongs to the RING-domain containing E3 ubiquitin ligase family. Its role is not well studied in cancer. Thus, studying the roles of IGF2BP2 and RNF13 could provide novel mechanisms implicated in ESCC. As shown in this study, similar findings on this chromosomal hot spot region were recently reported in lung squamous cell carcinoma [36], indicating that recurrent copy number amplifications in the 3q24-29 amplicon might play a major role in the development of squamous cell carcinomas.

### 3.4. Pathway-Level Alterations Involved in the Development of ESCC

**Figure 4:**
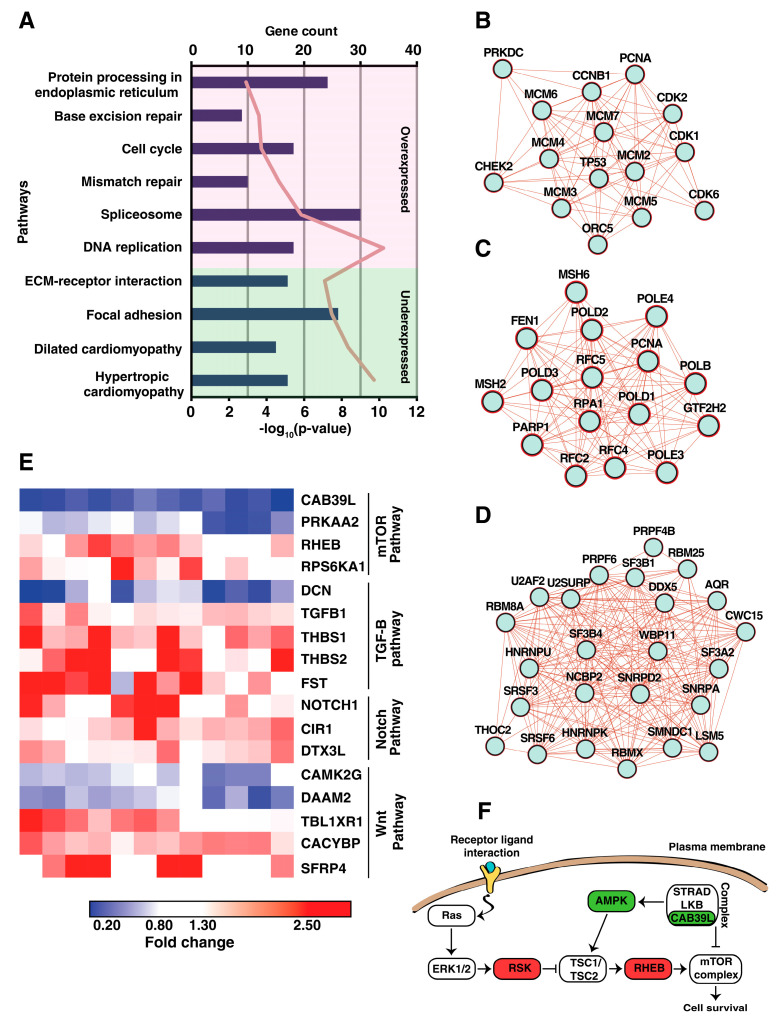
Functional characterization of the differentially expressed proteins in ESCC. (**A**) KEGG pathway enrichment analysis of the differentially expressed proteins performed using DAVID resource. Statistical significance of the enriched pathways was performed using Fisher’s exact test (*p*-Value ≤ 0.05). Protein–protein interaction network of dysregulated proteins involved in the (**B**) cell cycle, (**C**) DNA repair, and (**D**) spliceosome pathways. (**E**) Heat map visualization of the differentially expressed proteins across the 12 tumor samples which are involved in cancer-related pathways. The color strip indicates the fold change of the dysregulated protein, i.e., red color indicates overexpression, and blue color indicates the downregulation. (**F**) Mapping of the dysregulated proteins involved in the activation of the mTOR pathway in ESCC. Proteins highlighted in red are overexpressed, and those highlighted in green are downregulated in ESCC.

We then performed KEGG pathway analysis of differentially expressed proteins to identify functionally relevant pathways dysregulated in esophageal squamous cell carcinoma (Supplementary Table S5). Cell cycle, spliceosome, and DNA replication and repair pathways were significantly enriched among overexpressed proteins in ESCC (Figure 4A).

Overexpressed proteins associated with cell cycle include CCNB1, CDK1, CDK2, CDK6, PCNA, MCM complex, HDAC1, and PRKDC (Figure 4B). Several spliceosome-related proteins, including SRSF3, SRSF6, SF3B1, SF3A2, RBM8A, PRPF4, RBM25, and CTNNBL1, were shown to be overexpressed in multiple cancers [37,38,39,40] (Figure 4C). Several proteins involved in DNA repair pathways were overexpressed, including MSH2, MSH6, PARP1, FEN1, POLD1, and POLD2 (Figure 4D). Previous studies have reported increased mutagenesis and risk of metastasis due to overexpression of proteins involved in DNA repair pathways [41,42,43]. Downregulated proteins showed enrichment of focal adhesion, ECM-receptor interaction, and amino acid metabolism pathways such as tyrosine and histidine metabolism. Downregulated proteins involved in regulation of ECM-receptor interaction include ITGA5, ITGB1, TNXB, LAMB2, LAMA5, and HSPG2. Several ECM modifying enzymes were overexpressed in tumors, including serine proteases such as MMPs and HTRAs, suggesting distinct modification of ECM composition for tumor progression in ESCC.

Furthermore, we identified dysregulation of several proteins involved in key pathways related to tumor progression, such as mTOR, NOTCH, and TGFB, indicating their activation in ESCC (Figure 4E). In normal physiological conditions, responding to energy stress, STRAD/LKB/CAB39L complex activates AMPK, which further activates the TSC1/TSC2 complex, leading to negative regulation of the mTOR pathway [44]. We observed downregulation of CAB39L and PRKAA2, the catalytic subunits of AMPK. RPS6KA1 is a downstream effector molecule of MAPK/ERK pathway activation, which inhibits the TSC1/2 complex, and we identified the overexpression of RPS6KA1 (Figure 4F). RHEB, overexpressed in tumor tissues, is a GTP-binding protein positively regulating mTOR activation [45]. Differential expression of these proteins indicates the involvement of mTOR pathway as one of the tumorigenic mechanisms and targeting the mTOR pathway may provide therapeutic benefit in ESCC.

The Notch pathway is known to play a key role in squamous differentiation, and inactivating mutations in NOTCH1 were frequently observed in squamous cell carcinomas. We observed overexpression of NOTCH1 and other members of Notch pathway including CIR1, DTX3L, and HDAC1.

### 3.5. Differential Expression of Proteins Involved in ER Stress Response in ESCC

**Figure 5:**
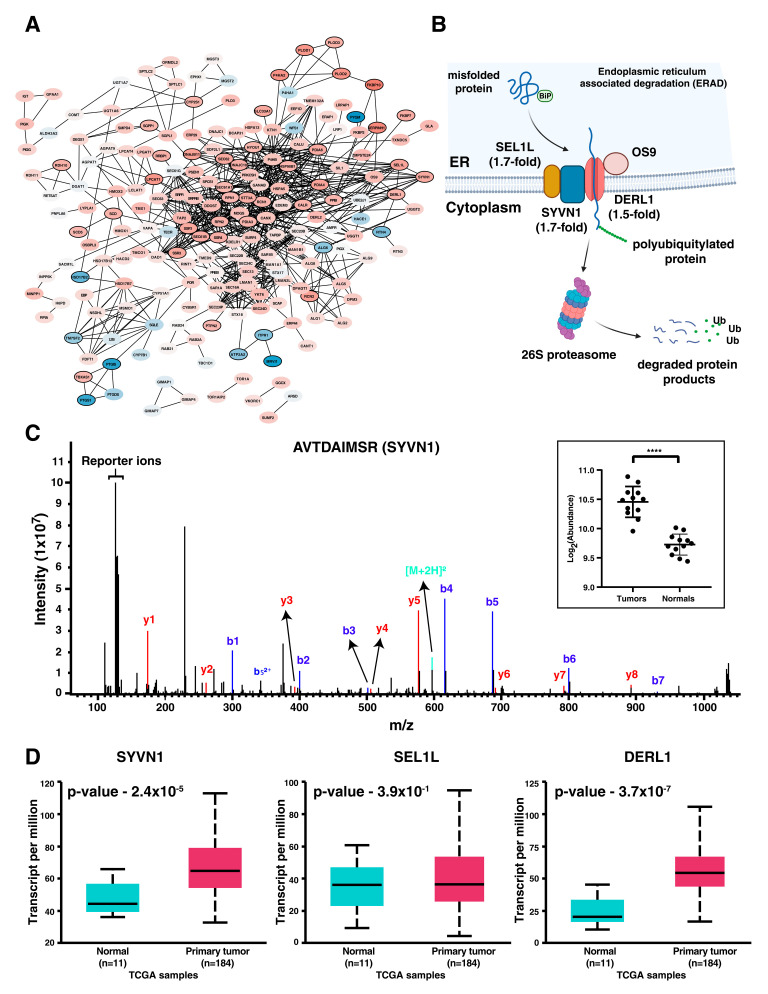
Enrichment of several ER-specific proteins among the differentially expressed proteins in ESCC. (**A**) Protein–protein interaction network of the proteins localized to endoplasmic reticulum indicates that several proteins were significantly overexpressed in ESCC. (**B**) Depiction of the ERAD process responsible for the clearance of misfolded/unfolded proteins in ER. Fold change of the proteins differentially expressed in ESCC is mentioned along with the protein. (**C**) Representative MS/MS spectrum of the peptide AVTDAIMSR belonging to SYVN1. The graph given in the inset represents the plotting of protein abundances across tumor and normal tissue samples (****—*p*-Value < 0.001). (**D**) Expression of SYVN1, SEL1L, and DERL1 in the RNAseq data of TCGA-ESCA cohort using UALCAN platform. SYVN1 and DERL1 were significantly overexpressed in tumors compared to normal tissue samples (*p*-Value < 0.05).

We identified overexpression of several proteins localized to the endoplasmic reticulum, including chaperons, transport channels, and endoplasmic reticulum-associated degradation (ERAD) machinery. The protein–protein interaction network of all the proteins localized to ER is represented in Figure 5A. ERAD is one of the crucial quality control mechanisms involved in maintaining ER homeostasis. It targets the misfolded/unfolded proteins and translocates them into the cytoplasm, promoting proteasomal degradation (Figure 5B). The SYVN1/SEL1L complex forms a major part of the ERAD machinery where synoviolin (SYVN1), a RING-domain containing E3 ubiquitin ligase, polyubiquitinates the substrates, and SEL1L is the adaptor protein responsible for the stability of SYVN1. We observed overexpression of both SYVN1 and SEL1L in our study (Figure 5C). Derlin (DERL1), another overexpressed protein belonging to ERAD pathway, forms the retrotranslocation channel interacting with the misfolded proteins and translocating them into the cytoplasm. SYVN1 and DERL1 were also significantly overexpressed in tumor tissues in the TCGA-ESCA dataset, indicating the significance of the overexpression of ERAD machinery in ESCC (Figure 5D).

In addition to proteins destined for ERAD, SYVN1 is also known to ubiquitinate non-canonical targets such as SIRT2, PTEN, and IGF-1R, promoting their degradation [46,47,48]. Notably, SYVN1 promoted the tumorigenic phenotype by regulating the ubiquitination and degradation of SIRT2 and PTEN in lung cancer and hepatocellular carcinoma [46,47]. SYVN1 was shown to inhibit apoptosis in renal tubular epithelial cells by mediating eIF2α ubiquitylation and degradation [49]. Thus, SYVN1 could be a novel therapeutic target, possibly playing a protective role in the development of ESCC.

## 4. Discussion

ESCC is a prevalent cancer with limited targeted therapeutic options. Deciphering molecular alterations associated with ESCC is important to identify biomarkers and therapeutic targets. In this study, we carried out unbiased global proteomic profiling to identify differentially expressed proteins in ESCC. In comparison with ESCCAtlas [50], a compendium of biomarkers cataloged by an extensive literature survey in ESCC, we identified several well-known differentially expressed proteins in common, indicating the validity of the findings in this study. For example, we identified the overexpression of several members of the matrix metalloproteinases family, including MMP9 [51], MMP13 [51,52], MMP12 [53], and MMP1 [54], which were previously shown as prognostic indicators in ESCC. Furthermore, TP63 is a transcription factor abundantly expressed in the proliferative basal layer of normal squamous epithelia. We found TP63 overexpression in esophageal tumors compared to normal tissues. Overexpression of TP63 was reported in other squamous cell carcinomas, including ESCC [55,56,57]. Interestingly, keratin 16 is another esophagus tissue-specific protein that we found overexpressed in esophageal cancer. It is not well studied in ESCC. It is a type I cytokeratin paired with keratin 6 (KRT6) in normal epithelia, and it was shown as a marker for hyperproliferative keratinocytes in psoriasis [58].

Enrichment analysis of functional pathways associated with ESCC revealed several important pathways among the overexpressed proteins, such as cell cycle, DNA replication, and spliceosome. A recent study on the proteomics and phosphoproteomics of tumor samples also indicated that these pathways are overexpressed in ESCC [59]. Overexpression of cell cycle components identified in this study includes cyclin-dependent kinases CDK6 and CDK4, which can be potential therapeutic targets in ESCC. Palbociclib, a CDK6 inhibitor, was recently approved by the FDA for treating breast cancer. In addition to the cell cycle, the enrichment of DNA replication and DNA repair pathways indicates the continuous proliferative demand of the tumor cells and the increased DNA repair activity necessary for the tumor cells to progress through the cell cycle. This change in replication kinetics and DNA repair activity could increase genomic instability, which is a hallmark of tumor progression [42]. These results indicate that the overexpression of DNA repair pathways may play a role in ESCC and needs to be studied further. We also identified overexpression of several proteins involved in spliceosome machinery, including SF3B1, SF3B4, and SRSF3. Studies have previously demonstrated the therapeutic efficacy of targeting the SF3B complex in cancer models [60,61,62], thus indicating the potential role of developing novel approaches for targeting spliceosome machinery in ESCC.

In contrast to the well-defined tumor-suppressive role of NOTCH1 in cancer, we identified overexpression of proteins involved in the Notch pathway, including NOTCH1. Higher expression of the NOTCH 1 intracellular domain (NICD) was previously shown in ESCC, compared to benign esophageal squamous epithelium and eosinophilic esophagitis. It is further associated with tumor grade and stage [63]. Overexpression of NOTCH1 was also reported in other squamous cell carcinomas such as HNSCC [64], cutaneous SCC [65] and oral SCC [66,67]. NOTCH1 is reported as both a tumor suppressor and an oncogene across squamous cell carcinomas [68]. Further characterization of NOTCH1 across large cohorts of ESCC may identify a subgroup of ESCC with an oncogenic role of NOTCH1, which can be treated with NOTCH1 pathway inhibitors. Our results also indicate activation of the mTOR pathway, a known oncogenic pathway involved in cell survival and proliferation. mTOR inhibition decreased the proliferation of ESCC cell lines [69] and showed similar results in an in vivo model, which further sensitized the tumor to cisplatin resistance [70,71]. A recent study by Campbell et al. on the multi-omic analysis of pan-squamous cell carcinomas also suggested the activation of the mTOR pathway [33]. Targeting the mTOR pathway could be an effective therapeutic strategy in ESCC.

We identified several novel differentially expressed proteins that can be evaluated for their potential therapeutic role in ESCC. For example, we identified two zinc channels, SLC39A7 (ZIP7) and SLC39A14 (ZIP14), among the top differentially overexpressed proteins in ESCC. These proteins maintain cytosolic Zn^2+^ levels by transporting zinc from ER reserves and the plasma membrane into the cell. As zinc is a cofactor for several enzymes and transcription factors in the cell, the zinc ion influx mediated by these channels could be involved in modulating several key cellular processes during tumor progression. For example, zinc release mediated by ZIP7 was previously reported as a hub for tyrosine kinase activation. Phosphorylation of ZIP7 was shown to drive cellular growth and proliferation pathways, including the mTOR pathway [72]. Utilizing the TCGA ESCC transcriptome data, we observed that ZIP7 expression is negatively correlated with the AMPK pathway, suggesting that overexpression of ZIP7 modulates the mTOR pathway activation, possibly through AMPK pathway regulation. Another novel protein identified in our study includes the overexpression of the SYVN1/SEL1L complex, part of the ERAD machinery, which plays an important role in ER quality control. SYVN1 is overexpressed by 1.5-fold in 8 out of 12 samples. SYVN1 is an E3 ubiquitin ligase that catalyzes ubiquitination of misfolded proteins within ER, thereby enabling its degradation and alleviating ER stress. SEL1L is overexpressed in 9 out of 12 samples in our study. It is an adaptor protein for SYVN1, enhancing its stability. This overexpression of ERAD machinery potentially relates to a pro-survival strategy for the cancer cells to clear misfolded proteins and reduce ER stress. The activity of SYVN1 is also reported beyond ER-resident proteins such as SIRT2 and PTEN, suggesting a non-canonical role for SYVN1. The role of SYVN1 needs to be studied in the future, and it can be evaluated as a potential therapeutic target in ESCC.

One of the main limitations of our study is the small sample size used for the quantitative proteomic analysis. Adjacent normal tissue used as control in this study could also pose several challenges. Normal tissue adjacent to the tumor displays an intermediate state between healthy and tumor, as shown by Aran et al. [73]. Despite these limitations, we identified several differentially expressed proteins that were previously reported in ESCC, which demonstrates the validity of these findings. Novel candidates identified in this study warrant further validation in a large cohort.

## 5. Conclusions

In this study, we identified several differentially expressed proteins in ESCC using a quantitative proteomics approach. We observed the overexpression of NOTCH1 and its pathway substrates. In addition, we found the overexpression of proteins involved in the ER stress response pathway. Preclinical studies targeting these pathways in ESCC are warranted to evaluate their potential clinical utility. Further validation of differentially expressed proteins in a large number of samples can yield potential biomarkers that could be used for diagnosing and monitoring ESCC.

## Data Availability

The mass spectrometry proteomics data have been deposited in the ProteomeXchange Consortium via the PRIDE [74] partner repository with the dataset identifier PXD036238.

## Supplementary Materials

The following supporting information can be downloaded at: https://www.mdpi.com/article/10.3390/cancers15133302/s1. Figure S1: (A) Sample correlation matrix displaying clustering of the tumor and normal tissue samples across the experiments, validating the inter-experimental normalization performed using internal reference scaling method. (B) Heatmap of the differentially expressed proteins (*p*-Value < 0.05, ANOVA) in tumors (T) compared to normals (N) between chewers (C), smokers (S), and tobacco non-users (N) in ESCC samples. Figure S2: Scatter plot of the differentially expressed proteins identified in this study with the transcript fold change from four published transcriptome studies on ESCC. Table S1: Clinical features of ESCC samples used for quantitative proteomic analysis. Table S2: List of all the proteins identified at 1% FDR cut-off in all three experiments. Table S3: List of loading points on the principal components for the proteins analyzed by PCA. Table S4: List of differentially expressed proteins in ESCC. Table S5: List of KEGG pathways enriched among the differentially expressed proteins in ESCC.
