# Supplementary figures and images for: Molecular Characterization of Esophageal Squamous Cell Carcinoma Using Quantitative Proteomics
Source: Cancers (Basel). 2023 Jun 23;15(13):3302. doi: 10.3390/cancers15133302 (PMC10340553; doi:10.3390/cancers15133302)

### Supplementary Figure S1A

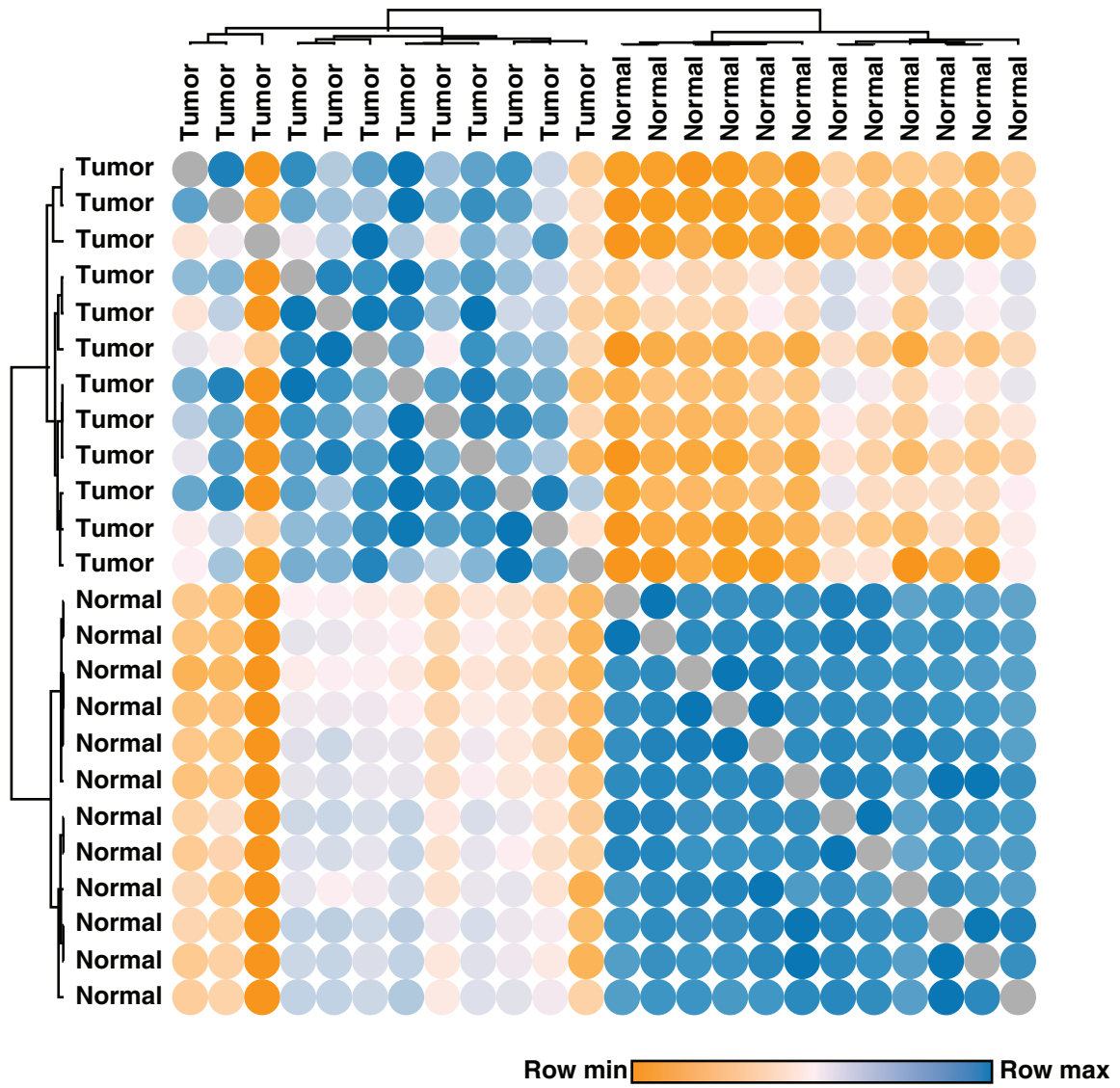

Supplement: Supplementary file 1 [file cancers-15-03302-s001.zip › Supplementary Figure S1A.pdf]

Supplementary Figure S1B

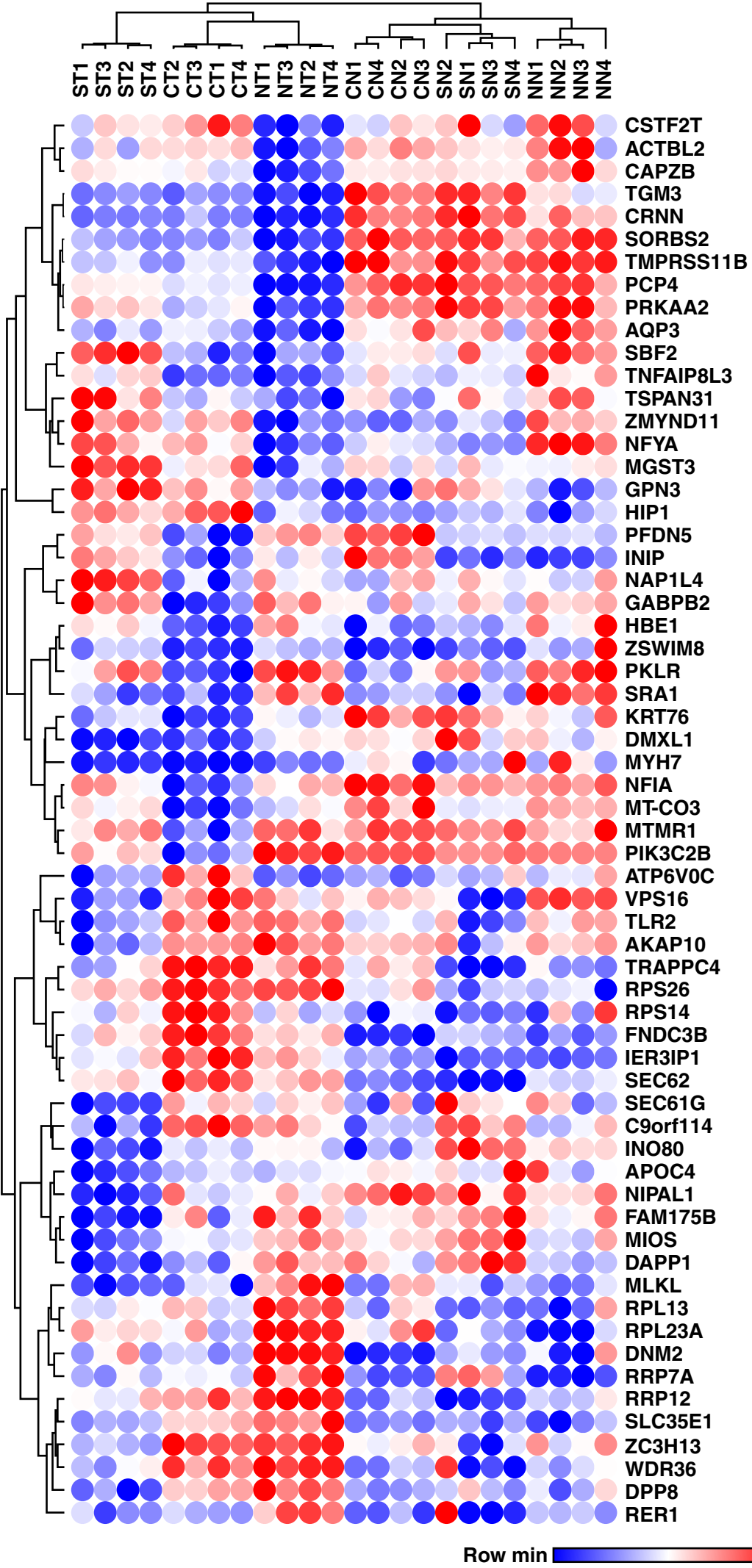

Row min Row max

Supplement: Supplementary file 1 [file cancers-15-03302-s001.zip › Supplementary Figure S1B.pdf]

Supplementary Figure S2

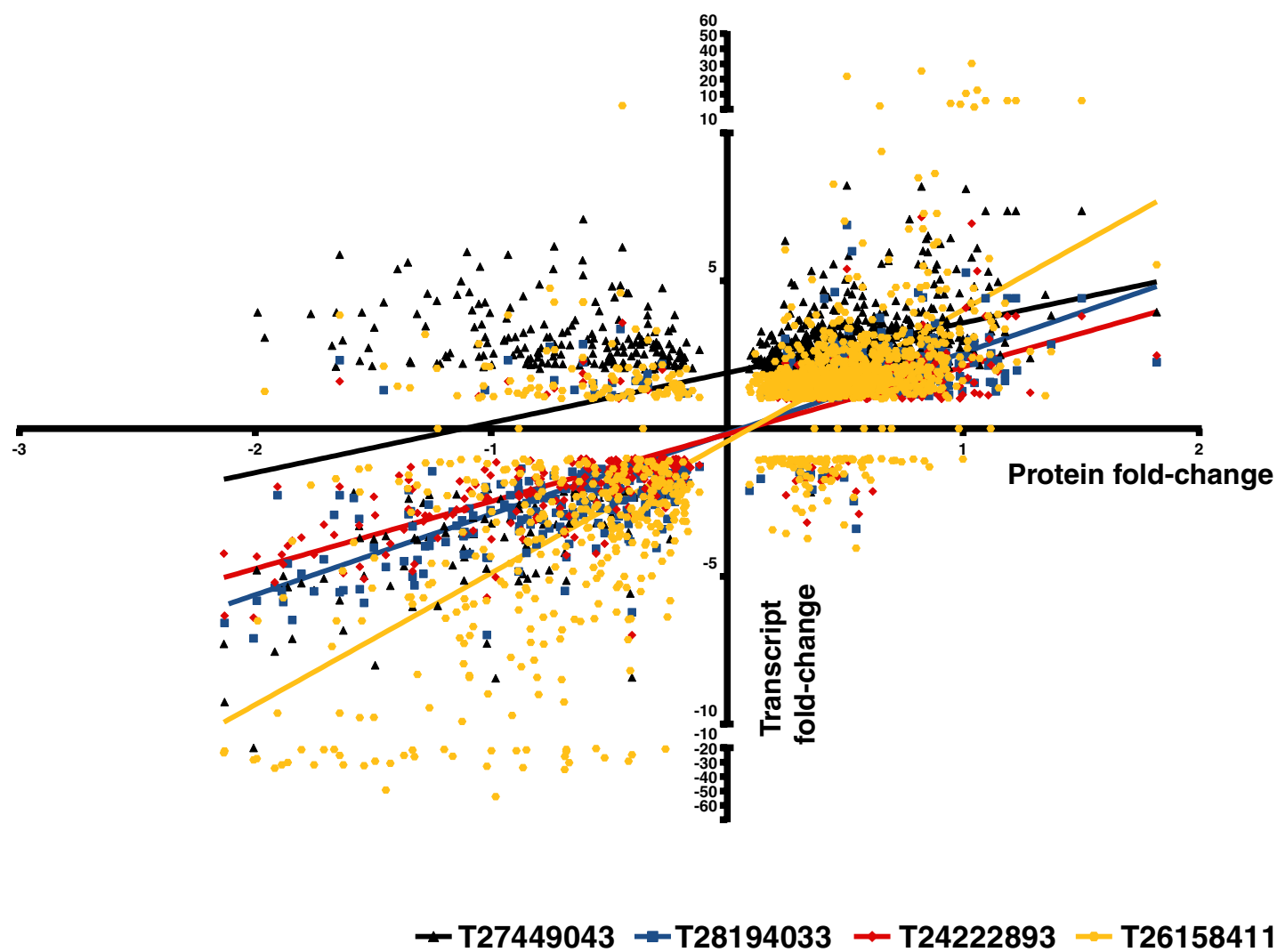

Supplement: Supplementary file 1 [file cancers-15-03302-s001.zip › Supplementary Figure S2.pdf]
